# Sex-specific COX-2/CREB/ER signaling underlies male susceptibility to pulmonary fibrosis in rheumatoid arthritis-associated interstitial lung disease

**DOI:** 10.3389/fimmu.2026.1753418

**Published:** 2026-05-20

**Authors:** Misong Kim, Kyoung-Soo Kim, Seung-Jae Hong, Jeehee Youn, Young Il Kim, Yeon-Ah Lee

**Affiliations:** 1 Department of Biomedical Science, Graduate School, Kyung Hee University, Seoul, Republic of Korea; 2 Department of Clinical Pharmacology and Therapeutics, School of Medicine, Kyung Hee University, Seoul, Republic of Korea; 3 Division of Rheumatology, Department of Internal Medicine, School of Medicine, Kyung Hee University, Seoul, Republic of Korea; 4 Department of Anatomy and Cell Biology, College of Medicine, Hanyang University, Seoul, Republic of Korea; 5 Medical Science Research Institute, Kyung Hee University Medical Center, Seoul, Republic of Korea

**Keywords:** COX-2, CREB, estrogen receptor, fibrosis, interstitial lung disease, rheumatoid arthritis, sex differences, SKG mouse

## Abstract

**Objectives:**

Rheumatoid arthritis is more prevalent in women than in men, but rheumatoid arthritis-associated interstitial lung disease occurs more frequently and with greater severity in men. To address this paradox, we investigated whether sex- and tissue-specific regulation of the cyclooxygenase-2/cyclic AMP-responsive element-binding protein/estrogen receptor axis contributes to the distinct rheumatoid arthritis-associated interstitial lung disease manifestations.

**Methods:**

Curdlan-induced SKG mice were used to assess sex-dependent disease manifestations. Arthritis severity was evaluated both clinically and histologically. Pulmonary inflammation and fibrosis were analyzed histologically, whereas gene and protein expression levels were assessed by quantitative reverse transcription polymerase chain reaction and western blotting.

**Results:**

Female mice developed earlier and more severe joint arthritis, whereas male mice exhibited greater pulmonary inflammation and fibrosis. These sex- and tissue-specific differences were supported by differential expression of fibrosis-related genes (fibronectin, collagen type I alpha 1 chain, vimentin, mucin-1, and E-cadherin). Lung tissues from males showed higher estrogen receptor α and estrogen receptor β expression, while joint estrogen receptor expression was predominantly higher in females, and no sex-related differences were observed in intestinal estrogen receptor expression. Consistent with estrogen receptor expression patterns, cyclooxygenase-2 and cyclic AMP-responsive element-binding protein expression were elevated in male lungs. Collectively, these findings suggest that sex- and organ-specific activation of the cyclooxygenase-2/cyclic AMP-responsive element-binding protein/estrogen receptor axis contributes to the inflammatory patterns in Rheumatoid arthritis-associated interstitial lung disease.

**Conclusion:**

Our findings suggest that cyclooxygenase-2/cyclic AMP-responsive element-binding protein/estrogen receptor pathway activation in male lungs may explain their increased tendency toward fibrotic remodeling, whereas enhanced estrogen receptor signaling in female joints may contribute to more severe arthritis. These observations raise the possibility that sex- and tissue-specific regulation of this signaling axis influences rheumatoid arthritis-associated interstitial lung disease pathogenesis and may inform future research on therapeutic strategies.

## 1 Introduction

Rheumatoid arthritis (RA) is a chronic autoimmune disease characterized primarily by synovial inflammation and joint destruction; however, it is also associated with extra-articular manifestations. Interstitial lung disease (ILD) is one of the most severe complications affecting 10–30% of patients and reducing survival, with a median life expectancy of only 3–7 years after diagnosis (1–4). Rheumatoid arthritis-associated interstitial lung disease (RA-ILD) is characterized by diverse histopathological patterns, most commonly usual interstitial pneumonia and nonspecific interstitial pneumonia, and its clinical course is highly variable, ranging from subclinical radiographic findings to rapidly progressive respiratory failure (3, 4). As the leading cause of non-articular mortality in RA, accounting for approximately 10–20% of all RA-related deaths, understanding the pathogenic mechanisms underlying RA-ILD is of considerable clinical importance. Established risk factors for RA-ILD include smoking, older age, autoantibody positivity, and male sex (5). Although RA is more common in women, RA-ILD occurs disproportionately in men with reported male-to-female ratios of up to 2:1 (6). This sex disparity suggests that sex hormone-related pathways influence pulmonary involvement in patients with RA.

Pulmonary fibrosis in RA-ILD results from dysregulated repair of alveolar epithelial injury under chronic inflammation, leading to excessive extracellular matrix deposition and progressive loss of lung function (7–9). Increasing evidence indicates that estrogen signaling contributes to lung fibrosis. Estrogen receptors, particularly estrogen receptor α (ER-α), have been implicated in myofibroblast differentiation and pro-fibrotic gene regulation, while estrogen receptor β (ER-β) may exert protective effects (10, 11). In idiopathic pulmonary fibrosis, ER-α expression is elevated, and antagonism of ER-α attenuates experimental fibrosis (10). These findings suggest that an imbalance in ER signaling may contribute to sex-specific fibrotic responses relevant to RA-ILD.

Estrogen receptor signaling intersects with pro-inflammatory pathways in which cytokines such as interleukin (IL)-1, IL-6, and tumor necrosis factor-alpha (TNF-α) induce cyclooxygenase-2 (COX-2), resulting in increased prostaglandin E_2_ production (12, 13). Through cAMP/protein kinase A-dependent signaling, COX-2-derived mediators may promote cyclic AMP-responsive element-binding protein (CREB) phosphorylation, which in turn can modulate ER transcriptional activity (14, 15). The COX-2/CREB/Estrogen receptor axis may therefore represent a candidate pathway linking chronic inflammation to sex-dependent fibrotic remodeling relevant to RA-ILD.

SKG mice, a T cell-driven autoimmune arthritis model, develop lung inflammation and fibrosis that reproduce selected features of human RA-ILD (16, 17). The SKG model carries a loss-of-function mutation in ZAP70 that allows autoreactive CD4^+^ T cells to escape thymic selection (16). Upon curdlan stimulation, these mice develop chronic autoimmune arthritis accompanied by progressive interstitial pneumonia, characterized by alveolar inflammation, fibrosis, and CD4^+^ T cell infiltration, including nonspecific interstitial pneumonia-like histopathological features that overlap with a subset of human RA-ILD (18). The model also shares certain serological features with human RA, including the production of autoantibodies such as rheumatoid factor (19). Furthermore, the Th17-driven inflammatory milieu, marked by elevated IL-17A and Granulocyte-Macrophage Colony-Stimulating Factor, supports the utility of this model for investigating pathways potentially relevant to RA-ILD pathogenesis, although it does not fully replicate the complexity of human disease (20). Using this model, we investigated sex- and tissue-specific differences in ER expression and upstream COX-2/CREB activation. We hypothesized that preferential activation of the COX-2/CREB/ER pathway in male lungs may contribute to increased susceptibility to fibrotic remodeling in this murine model of RA-ILD-like disease. Accordingly, our aim was to identify sex- and tissue-specific signaling patterns associated with divergent joint and lung phenotypes, and to determine whether these findings may provide mechanistic insight into the male predominance of pulmonary fibrosis observed in human RA-ILD.

## 2 Materials and methods

### 2.1 Mice

SKG mice were originally developed by Dr. S. Sakaguchi (Osaka University, Japan) and were kindly provided by Dr. Jeehee Youn (Hanyang University, Seoul, Republic of Korea) for use in this study (19). Eight-to-nine-week-old SKG mice were maintained under specific pathogen-free conditions at the Institute of Laboratory Animals, Kyung Hee University Hospital, Gangdong, Republic of Korea. All animal experiments were conducted in accordance with the guidelines for animal care and approved by the Animal Experimentation Committee of Kyung Hee University Hospital, Gangdong (KHNMC AP 2022-010).

### 2.2 Induction of arthritis and ILD in SKG mice

Disease was induced following established procedures (21). A 3 mg dose of curdlan (Wako, Osaka, Japan, 032-09902) was selected to reliably induce both arthritis and lung fibrosis in SKG mice. Briefly, curdlan suspended in 0.2 mL PBS (Welgene, Gyeongsan, Republic of Korea, LB004-01) was injected intraperitoneally at weeks 0 and 2. A total of 27 mice (14 females and 13 males) received curdlan, whereas 10 control mice (5 females and 5 males) received PBS only. Arthritis severity was monitored weekly for 21 weeks and evaluated by three independently trained investigators blinded to group allocation. Clinical arthritis was scored for each paw according to the criteria summarized in **Table 1**. The total arthritis score for each animal was obtained by summing the scores of all four paws (22).

**Table 1:** Clinical arthritis scoring system.

Score	Clinical criteria
0	Normal
1	Swelling of one finger joint
2	Mild swelling of the wrist or ankle
3	Moderate swelling of the wrist or ankle
4	Severe swelling of the wrist or ankle

### 2.3 Anesthesia, euthanasia, and tissue collection

Mice were anesthetized with isoflurane (5% induction, 2% maintenance, Hana Pharm, Seoul, Republic of Korea) using a nose cone. Blood samples were collected via cardiac puncture under deep anesthesia. Euthanasia was then performed by gradual-fill CO_2_ inhalation (30–70% of the chamber volume/min) in accordance with American Veterinary Medical Association guidelines and institutional approval. To ensure complete euthanasia, animals were maintained in the chamber for at least 1 minute following respiratory arrest. The death of each animal was confirmed by the absence of a heartbeat and the lack of response to a firm toe pinch before proceeding with tissue collection. Lungs and paws were subsequently harvested and perfused with PBS via the trachea to remove blood. ILD was histologically confirmed at 21 weeks of age.

### 2.4 Histologic evaluation

Tissues were fixed with 4% formaldehyde (ByLabs, Hwaseong, Gyeonggi-do, Republic of Korea, P 0117CD). Paws were decalcified (>24 h), and paw and lung tissues were paraffin-embedded and sectioned at 4 μm. Sections were dewaxed in xylene (Duksan Pure Chemicals, Ansan, Gyeonggi-do, Republic of Korea, 001_00314) and rehydrated through a graded ethanol series. To evaluate inflammation, sections were stained with hematoxylin and eosin (H&E: Leica Biosystems, Richmond, IL, USA, 3802098). The severity of inflammation in paw and lung tissues was graded on a scale of 0–4, as detailed in **Table 2**. Additionally, masson’s trichrome staining (EMS, Hatfield, PA, USA, 26367-Series) was performed to assess lung fibrosis. Fibrosis was quantified using a modified Ashcroft scoring system (grades 0–4), with the specific criteria summarized in **Table 2** (23, 24). For immunohistochemical analyses, sections were incubated with anti-ER-α (Santa Cruz, Dallas, TX, USA, sc-71064) or anti–ER-β (Santa Cruz, Dallas, TX, USA, sc-390243), and detection was performed using 3,3′-diaminobenzidine staining (Dako, Carpinteria, CA, USA, K3468). At least 10 sections per animal were analyzed using LEICA Qwin V3 software for quantification.

**Table 2:** Histopathological scoring systems for inflammation and fibrosis.

Category	Score	Description
Inflammation	0	None
	1	Mild
	2	Mild to moderate
	3	Moderate
	4	Severe
Lung Fibrosis	0	Normal lung architecture
	1	Minimal fibrous thickening of alveolar or bronchiolar walls
	2	Moderate thickening of walls without obvious damage to lung architecture
	3	Increased fibrosis with definite damage to lung structure and formation of fibrous bands or small fibrous masses
	4	Severe distortion of the structure and large fibrous areas

### 2.5 RT-qPCR Analysis

Total RNA was extracted from mouse lung tissues using TRIzol reagent (Invitrogen, Carlsbad, CA, USA, 15596018) according to the manufacturer’s protocol. First-strand cDNA was synthesized from 1 μg of total RNA using oligo (dT) primers (K1622, Thermo Fisher, Waltham, MA, USA). Quantitative real-time PCR was performed using a StepOnePlus Real-Time PCR System with Power SYBR Green PCR Master Mix (Applied Biosystems, Waltham, MA, USA, 4367659). Each 20 μL reaction mixture contained 1 μL cDNA, 10 μL Master Mix, 2 μL primers (**Table 3**), and 7 μL PCR-grade water. The cycling conditions were as follows: 95 °C for 10 min, followed by 40 cycles of 95 °C for 15 s and 60 °C for 1 min. Relative mRNA expression levels were calculated using the ΔΔCt method and normalized to β-actin.

**Table 3:** Primer sequences used for RT-qPCR.

Gene	Primer sequences	Product size (bp)	Gene bank
IL-6	5′-TACCACTTCACAAGTCGGAGGC-3′	115	NM_031168.2
	5′-CTGCAAGTGCATCATCGTTGTTC-3′		
TNF-α	5′-CCTGTAGCCCACGTCGTAG-3′	147	NM_013693.3
	5′-GGGAGTAGACAAGGTACAACCC-3′		
β-actin	5′-GGCTGTATTCCCCTCCATCG-3′	153	NM_007393.5
	5′-CCAGTTGGTAACAATGCCATGT-3′		

RT-qPCR, quantitative reverse transcription polymerase chain reaction; IL-6, interleukin-6; TNF-α, tumor necrosis factor-α

### 2.6 Western blot analysis

Proteins were extracted from frozen lung and intestinal tissues using a radioimmunoprecipitation assay buffer(Thermo Scientific, Rockford, IL, USA, 89900) supplemented with protease and phosphatase inhibitors. Protein concentrations were determined by the bicinchoninic acid assay (Thermo Scientific, Rockford, IL, USA, 23225). Equal amounts of protein (10 µg per lane) were separated by sodium dodecyl sulfate-polyacrylamide gel electrophoresis and transferred onto nitrocellulose membranes. Membranes (Cytiva, Dassel, Germany, 10600002) were blocked with 5% (w/v) non-fat dry milk in Tris-buffered saline containing 0.05% Tween-20 and incubated with primary antibodies against fibronectin, collagen type I alpha 1 chain, vimentin, mucin-1, E-cadherin, ER-α, ER-β, COX-2, CREB, phosphorylated CREB (p-CREB), and β-actin (**Supplementary Table 1**). After washing, membranes were incubated with horseradish peroxidase–conjugated secondary antibodies (Abcam, Cambridge, anti-rabbit, ab97051; anti-mouse, ab6789). Signals were detected using ECL Substrate (Thermo Scientific, Rockford, IL, USA, 34095) and visualized using an Amersham Imager 600. Band intensities were quantified using ImageJ software (v1.44) and normalized to β-actin as a loading control.

### 2.7 Statistical analysis

Data were analyzed using GraphPad Prism v8.0 (GraphPad Software, San Diego, CA, USA). Mixed-effects models were used for intergroup and intragroup comparisons. As the variables were not normally distributed, the Kruskal–Wallis test was applied, followed by Dunn’s multiple comparisons. Results are expressed as mean ± standard deviation. Statistical significance was set at *P* < 0.05.

## 3 Results

### 3.1 Differential arthritis severity between male and female SKG mice

**Figure 1:**
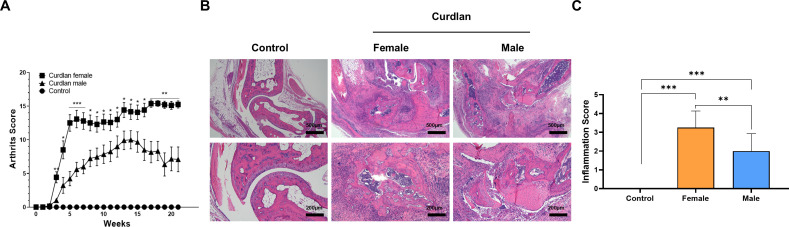
Sex differences in arthritis severity in curdlan-induced SKG mice. **(A)** Clinical arthritis scores assessed weekly for 21 weeks. Female curdlan-treated mice exhibited earlier disease onset and higher arthritis severity than male mice. **(B)** Representative H&E-stained joint sections showing normal architecture in control mice and synovial hyperplasia, inflammatory infiltration, and cartilage destruction in curdlan-treated groups, with more extensive pathology in females. Scale bar = 500 μm (40x magnification) and 200 μm (100x magnification). **(C)** Quantitative histological inflammation scores confirmed significantly greater joint inflammation in female mice compared with males. Data presented as mean ± SD. *P < 0.05; **P < 0.01; ***P < 0.001. H&E, hematoxylin and eosin; SD, standard deviation.

To examine sex differences in arthritis severity, male (n = 13) and female (n = 14) SKG mice were intraperitoneally injected with curdlan (3 mg), whereas controls (n = 10) received PBS. Joint swelling and deformities were evaluated weekly for 21 weeks. As shown in **Figure 1A**, arthritis developed earlier in female mice (week 3) than in male mice (week 4) and progressively worsened over time. By week 21, female mice exhibited markedly higher arthritis scores compared with males (15.3 ± 1.5 vs. 4.8 ± 4.9, *P* < 0.01). Histological analysis confirmed severe synovial hyperplasia, inflammatory infiltration, and cartilage destruction in the curdlan-treated groups, with more extensive damage observed in females (**Figure 1B**). Quantitative histological scoring also revealed inflammation scores that were higher in female than in male mice (3.3 ± 0.9 vs. 2.0 ± 0.9, *P* < 0.01, **Figure 1C**). These findings indicate that curdlan-induced arthritis is more severe in females than in males.

### 3.2 Sex-dependent differences in pulmonary inflammation and fibrosis

**Figure 2:**
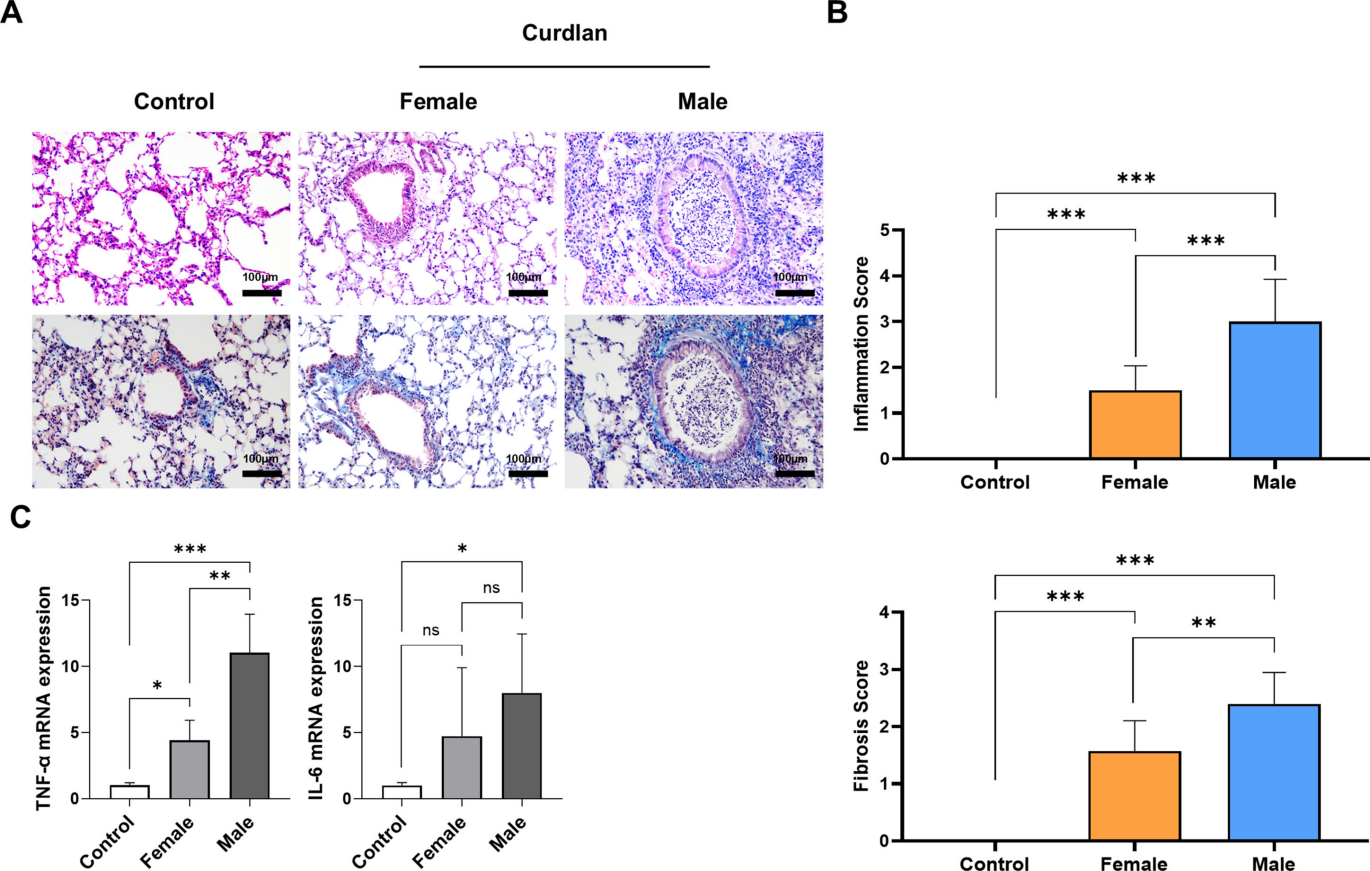
Sex dependent pulmonary inflammation and fibrosis in curdlan-induced SKG mice. **(A)** Representative images of H&E (upper panels) and masson’s trichrome (lower panels) stained lung sections. Male mice show extensive inflammatory infiltration and peribronchial fibrosis (blue-stained areas), whereas females exhibit relatively preserved lung architecture. Scale bar = 100 μm. Magnification = 200x. **(B)** Histological scoring of pulmonary inflammation and fibrosis (Ashcroft score) revealing greater severity in male compared to female mice. **(C)** RT-qPCR analysis of pro-inflammatory cytokine mRNA in lung tissues. TNF-α expression was higher in males. Data presented as mean ± SD. ns, not significant; *P < 0.05; **P < 0.01; ***P < 0.001. Abbreviations: H&E, hematoxylin and eosin; RT-qPCR, reverse transcription quantitative polymerase chain reaction; TNF-α, tumor necrosis factor-α; IL-6, interleukin-6; SD, standard deviation.

To evaluate sex-based differences in lung involvement, lung sections were examined using H&E and masson’s trichrome staining (**Figure 2A**). Male mice exhibited extensive interstitial and alveolar inflammatory infiltration, along with prominent peribronchial collagen deposition. In contrast, female mice showed more preserved alveolar structures with only moderate inflammatory changes. Histological scoring confirmed that pulmonary inflammation and fibrosis levels were significantly higher in males than in females (**Figure 2B**; Inflammation score: 3.0 ± 0.9 vs. 1.5 ± 0.5, *P* < 0.001; Fibrosis score: 2.4 ± 0.5 vs. 1.6 ± 0.5, *P* < 0.01) RT-qPCR further demonstrated increased pro-inflammatory cytokine expression in the lungs of male mice. TNF-α mRNA was 2.5-fold higher in males than in females (*P* < 0.01), while IL-6 expression showed a non-significant upward trend (**Figure 2C**). These results indicate that pulmonary inflammation was more severe in male mice with RA-ILD.

### 3.3 Increased fibrosis-related protein expression in male lungs

**Figure 3:**
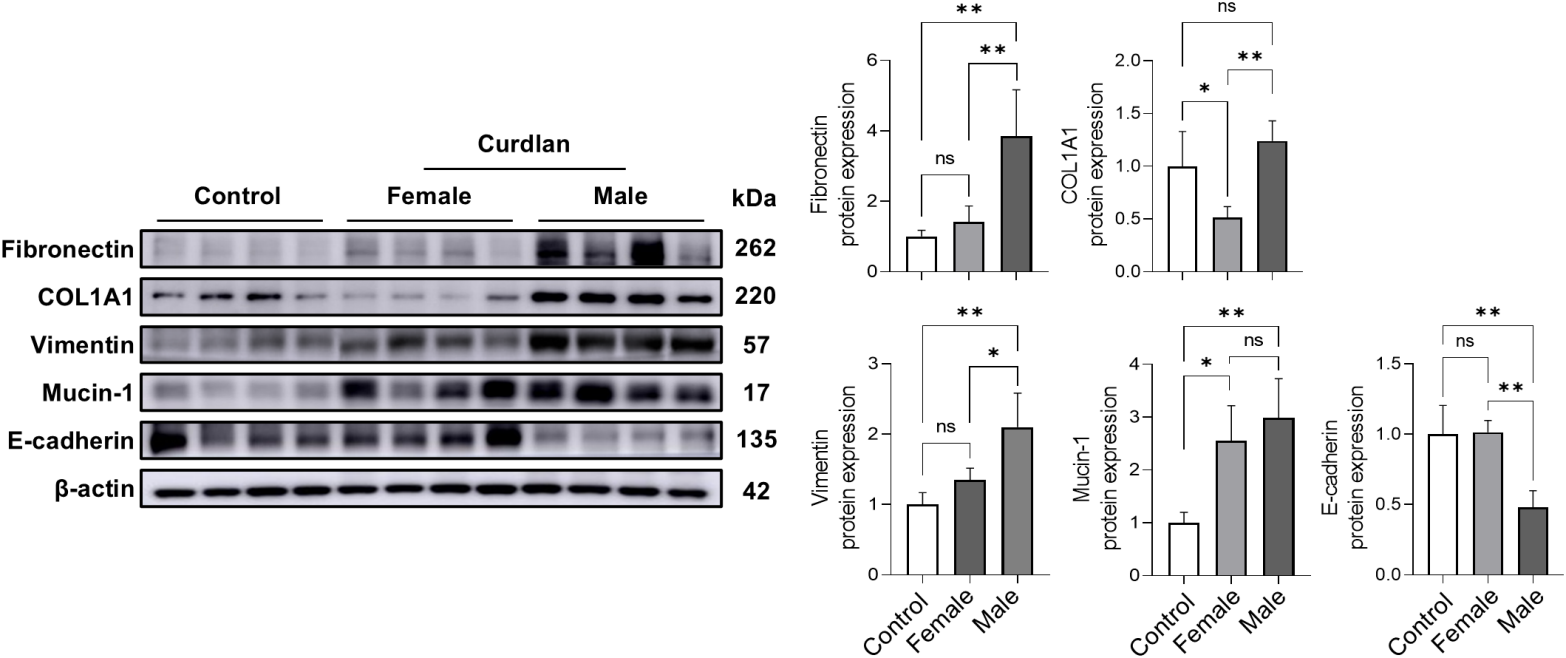
Increased fibrosis-related protein expression in male lungs. Western blot analysis with densitometric quantification of fibrosis-associated proteins in lung tissues. Male mice displayed higher expression of fibronectin, COL1A1, vimentin, and mucin-1 compared with female mice, whereas E-cadherin expression was reduced. Data are presented as mean ± SD. ns, not significant; *P < 0.05; **P < 0.01; ***P < 0.001. Abbreviations: COL1A1, collagen type I alpha 1 chain; SD, standard deviation.

To assess fibrotic remodeling, lung tissues were analyzed for fibrosis-associated proteins using western blotting (**Figure 3**). Male mice displayed significantly higher expression levels of fibronectin (2.7-fold), collagen type I alpha 1 chain (2.4-fold), vimentin (1.6-fold), and mucin-1 (1.2-fold) than female mice. In contrast, E-cadherin levels were markedly reduced in male lungs, indicating epithelial–mesenchymal transition, a process closely linked to fibrosis. These results suggest that male RA-ILD mice are more prone to fibrotic remodeling than female mice.

### 3.4 Upregulation of estrogen receptors and COX-2/CREB pathway in male lungs

**Figure 4:**
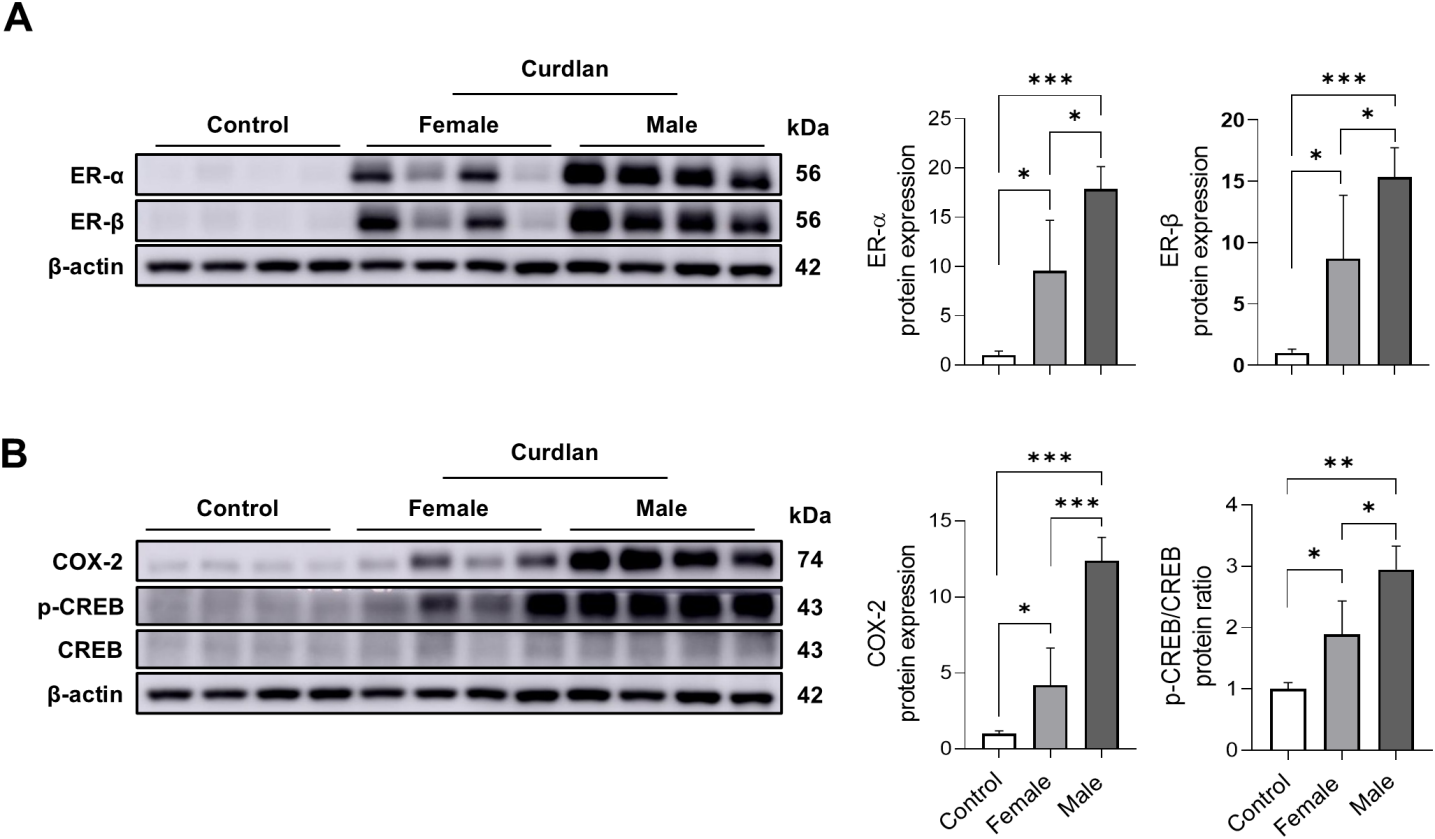
Enhanced estrogen receptor (ER) expression and COX-2/CREB pathway activation in male lungs. **(A)** Western blot analysis of ER-α and ER-β showed higher expression in male lung tissues compared with those in female lung tissues. **(B)** Expression of COX-2 and p-CREB was markedly increased in male lung samples. Data are presented as mean ± SD. ns, not significant; *P < 0.05; **P < 0.01; ***P < 0.001. Abbreviations: ER, estrogen receptor; COX-2, cyclooxygenase-2; CREB, cAMP response element-binding protein; p-CREB, phosphorylated CREB; SD, standard deviation.

Estrogen receptors (ER-α and ER-β) are known to influence fibrosis (25–27). Western blot analysis revealed significantly higher ER-α and ER-β expression in male lungs compared with that in female lungs (**Figure 4A**). To explore this mechanism, we examined the upstream COX-2/CREB signaling (28). COX-2 expression was increased by 12.4-fold versus controls and 3.0-fold compared to females, whereas p-CREB/CREB ratios were elevated by 2.9- and 1.6-fold, respectively (**Figure 4B**). These findings indicate that the COX-2/CREB/ER signaling axis is preferentially activated in male lungs, potentially contributing to the sex-specific susceptibility to pulmonary fibrosis.

### 3.5 Sex- and tissue-specific ER expression patterns

**Figure 5:**
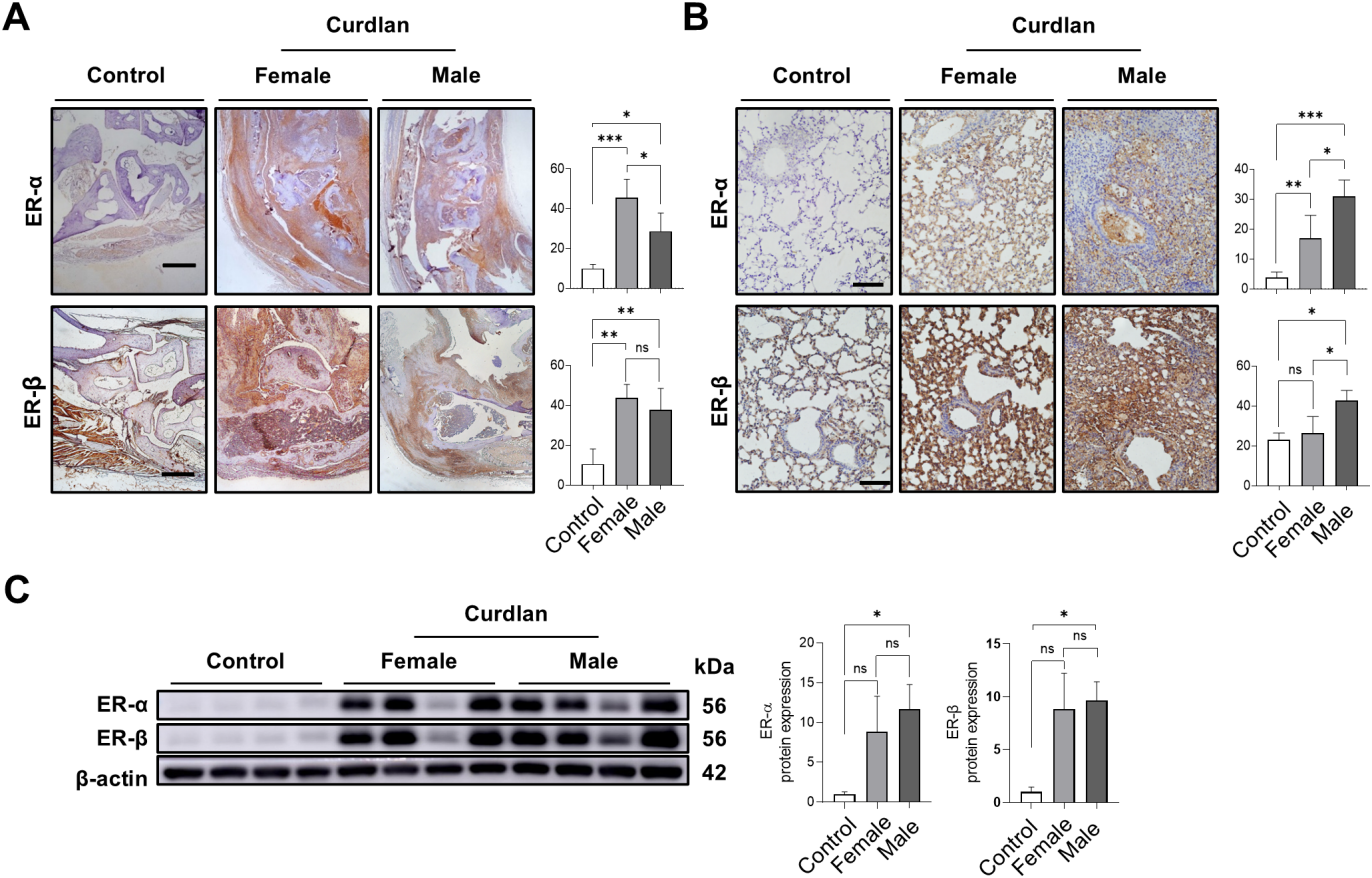
Sex- and tissue-specific expression of estrogen receptors (ERs). **(A)** Immunohistochemistry of paw tissues showed higher ER-α and ER-β expression in females compared with males. **(B)** Immunohistochemistry of lung tissues revealed higher ER-α and ER-β expression in males compared with females. Scale bars = 100 μm. **(C)** Western blot analysis of intestinal tissues indicated no significant sex-dependent differences in ER-α or ER-β expression. Data are presented as mean ± SD. ns, not significant; *P < 0.05; **P < 0.01; ***P < 0.001. Abbreviations: ER, estrogen receptor; SD, standard deviation.

To further evaluate tissue-specific differences, ER expression was assessed using immunohistochemistry (**Figure 5**). In paw tissues, ER-α expression was significantly higher in females than in males (45.7% vs. 28.7% stained area, *P* < 0.05), while ER-β expression was also higher in females, although this difference was not statistically significant (43.7% vs. 37.8%) (**Figure 5A**). In contrast, lung tissues showed the opposite trend, ER-α and ER-β expression levels were both significantly elevated in males (31.0% and 42.7%, respectively) compared with those in females (17.0% and 26.3%) (**Figure 5B**). These findings demonstrate sex- and tissue-specific ER regulation, with dominant female expression in the joints and predominantly male expression in the lungs.

### 3.6 No sex-based differences in intestinal ER expression

To determine whether these differences were systemic, ER expression was analyzed in intestinal tissues by western blotting (**Figure 5C**). No significant sex-based differences were observed in ER-α or ER-β expression, indicating that sex-specific ER regulation is tissue-specific rather than systemic, and may contribute to distinct pathological manifestations in RA-ILD.

## 4 Discussion

This study suggests that disease manifestations in the curdlan-induced SKG model differ according to sex. Female mice developed earlier onset and more severe arthritis, whereas male mice exhibited more pronounced pulmonary inflammation and fibrosis. These divergent phenotypes were associated with tissue-specific differences in ER, COX-2, and cyclic AMP-responsive element-binding protein signaling, supporting the possibility that sex-specific molecular programs may contribute to distinct inflammatory and fibrotic outcomes in this model.

Given that the SKG model effectively mimics the symmetrical inflammation and bone erosion of distal small joints seen in human RA, our analysis focused on the paw and ankle joint (29, 30). In this model, female mice developed earlier and more severe arthritis than male mice, which is consistent with the higher prevalence of RA in women. Clinical and histological assessments confirmed more pronounced synovial inflammation and cartilage destruction in females, accompanied by higher ER-α and ER-β expression in joint tissues. These findings align with reports in SKG and TNF-α transgenic models showing stronger arthritis phenotypes in females (31, 32). Mechanistically, ER signaling has been reported to enhance pro-inflammatory gene expression in synovial fibroblasts and immune cells, and ER-α activation can amplify NF-κB and AP-1 pathways that promote inflammatory cytokine production (33–35). Although these downstream mediators were not directly measured in joint tissue in the present study, the increased joint ER expression in female mice is consistent with enhanced local inflammatory responsiveness and may partly explain the more severe arthritic phenotype.

Conversely, pulmonary inflammation and fibrosis were more pronounced in male mice, as evidenced by higher inflammation/fibrosis scores and greater collagen deposition. At the molecular level, this was characterized by higher TNF-α expression and a marker profile including increased fibronectin, collagen type I alpha 1 chain, vimentin, and mucin-1, alongside decreased E-cadherin, reflecting the induction of epithelial-to-mesenchymal transition and extensive fibrotic remodeling. Importantly, both ER-α and ER-β were upregulated in male lungs. This finding differs somewhat from the prevailing view that ER-α is primarily associated with profibrotic remodeling, whereas ER-β may exert protective or counter-regulatory effects (10, 36). However, this difference may be explained by experimental context. Previous studies showing reduced ER-β expression were based mainly on epithelial cell-dominant systems or end-stage fibrotic tissue, whereas our study examined whole-lung tissue during active inflammatory and fibrotic remodeling (37, 38). Therefore, the increase in ER-β observed in our model may not indicate a directly profibrotic role, but instead may reflect a cell type-specific or compensatory response within the injured lung microenvironment. Male lungs also displayed marked upregulation of COX-2 and CREB phosphorylation, supporting a model in which COX-2-derived prostaglandins activate cAMP/Protein kinase A-CREB signaling, which in turn enhances ER transcriptional activity (39, 40). Hyperactivation of the COX-2/CREB/ER axis may create a feed-forward loop linking inflammation to aberrant repair, thereby driving male-predominant pulmonary fibrosis in RA-ILD.

In contrast, no sex-dependent differences were observed in intestinal ER expression, supporting the idea that these mechanisms are tissue specific rather than systemic. Organ selectivity in hormone receptor regulation has been observed in other contexts and is influenced by local chromatin accessibility and cytokine environments (41, 42). In RA, this tissue specificity may explain why the same systemic autoimmune milieu yields divergent outcomes in the joints and lungs.

Several limitations of this study warrant consideration. First, while the curdlan-induced SKG model reproduces key features of human RA-ILD, it primarily reflects a nonspecific interstitial pneumonia-like inflammatory setting, which may not fully capture the clinical complexity of human disease, including the prominence of usual interstitial pneumonia patterns, diverse genetic backgrounds, and environmental exposures. Second, as this was an observational study, definitive causality could not be established, and the lack of systemic hormone measurements and specific mediators such as transforming growth factor-β, C-X-C motif chemokine ligand 13, and Matrix metalloproteinase-7 limits a comprehensive characterization of the systemic versus local fibrotic milieu. Finally, the use of a fixed curdlan dose without sex-stratified dose-sensitivity analysis may have overlooked potential sex-dependent differences in responsiveness. Despite these constraints, our findings provide critical hypothesis-generating observations, identifying the COX-2/CREB/ER axis as a potentially significant pathway that merits further validation in human synovial and lung tissues.

## 5 Conclusion

This study suggests that sex-related differences in COX-2/CREB/ER signaling are tissue specific in this murine model of RA-ILD. Female mice exhibited more severe arthritis with enhanced ER expression in the joints, whereas male mice displayed heightened pulmonary inflammation and fibrosis associated with the upregulation of ERs, COX-2, and CREB. No sex-based differences were detected in the intestinal tissues. Taken together, these findings support the possibility that aberrant, sex- and tissue-specific regulation of the COX-2/CREB/ER axis may contribute to the divergence between articular and pulmonary manifestations. Although the present study does not establish causality, it provides a biologically plausible framework for understanding male-predominant fibrotic lung involvement in this murine model and supports further investigation of this pathway in human RA-ILD.

## Data Availability

The raw data supporting the conclusions of this article will be made available by the authors, without undue reservation.
