# Supplementary material for: Sex-specific COX-2/CREB/ER signaling underlies male susceptibility to pulmonary fibrosis in rheumatoid arthritis-associated interstitial lung disease
Source: Front Immunol. 2026 May 20;17:1753418. doi: 10.3389/fimmu.2026.1753418 (PMC13230087; doi:10.3389/fimmu.2026.1753418)
Supplement: Supplementary file 1 [file Table1.docx]

Supplementary Material

# Supplementary Table S1. Antibody list used for Western blot

| 1st antibody | Company | Catalogue Number | Host species | Dilution |
| --- | --- | --- | --- | --- |
| Fibronectin | Abcam | ab2413 | Rabbit | 1:1,000 |
| COL1A1 | Cell Signaling Technology | 72026 | Rabbit | 1:500 |
| MUC-1 | Abcam | ab109185 | Rabbit | 1:3,000 |
| E-Cadherin | Cell Signaling Technology | 3195 | Rabbit | 1:1,000 |
| Vimentin | Cell Signaling Technology | 5741 | Rabbit | 1:1,000 |
| ER-α | Santa Cruz | sc-71064 | Mouse | 1:250 |
| ER-β | Santa Cruz | sc-390243 | Mouse | 1:500 |
| Cox-2 | Cell Signaling Technology | 12282 | Rabbit | 1:1,000 |
| CREB | Cell Signaling Technology | 9197 | Rabbit | 1:1,000 |
| P-CREB | Cell Signaling Technology | 9198 | Rabbit | 1:1,000 |
| β-Actin | Cell Signaling Technology | 4970 | Rabbit | 1:3,000 |
| β-Actin | Sigma | A1978 | Mouse | 1:5,000 |

COL1A1, Collagen type I α 1 chain; MUC-1, Mucin 1; ER-α, Estrogen receptor α; ER-β, Estrogen receptor β; Cox-2, Cyclooxygenase-2; P-CREB, Phosphorylated cAMP response element-binding protein.
